# Metronomic chemotherapy in pediatric neuroblastoma

**DOI:** 10.1002/pdi3.2512

**Published:** 2024-12-10

**Authors:** Yi Jiang, Chengjun Xi, Chao Yang

**Affiliations:** ^1^ Department of Surgical Oncology National Clinical Research Center for Child Health and Disorders Ministry of Education Key Laboratory of Child Development and Disorders China International Science and Technology Cooperation Base of Child Development and Critical Disorders Children's Hospital of Chongqing Medical University Chongqing China

**Keywords:** clinical trial and review, metronomic therapy, neuroblastoma, pediatric

## Abstract

Metronomic chemotherapy (MC) is an innovative therapeutic approach that involves the chronic administration of low doses of chemotherapy agents. This strategy aims to sustain prolonged and active plasma levels of drugs, which can result in favorable tolerability. MC has shown promise in the treatment of hematologic and solid tumors, including high‐risk neuroblastoma and relapsed/refractory (R/R) neuroblastoma. In the contemporary management of neuroblastoma, MC stands as a viable maintenance therapy for newly diagnosed patients lacking access to autologous stem cell transplantation or immunotherapy, particularly in resource‐constrained regions. Furthermore, it serves as a pragmatic alternative for individuals intolerant to intensified regimens or undergoing palliative care for R/R neuroblastoma. Nevertheless, the quest for the optimal MC regimen persists, necessitating comprehensive investigations to delineate standardized protocols. Moreover, the identification of potential biomarkers or prognostic indicators assumes paramount significance in refining MC strategies for future breakthroughs in this domain. This review embarks on a comprehensive exploration of MC in neuroblastoma, offering insights into its historical underpinnings, diverse applications, adverse effect and future prospects, endeavoring to enrich our understanding of MC role in neuroblastoma management.

## 1 INTRODUCTION

Metronomic chemotherapy (MC) entails the chronic administration of chemotherapy at low doses, maintaining sustained and active plasma levels of drugs over an extended period. This approach offers improved tolerability and represents a promising therapeutic strategy for both solid and hematologic tumors. The concept was first introduced in the year 2000 by Kerbell^1^ and Hanahan^2^ in two separate articles. In contrast to the conventional chemotherapy regimens predicated upon the administration of maximal doses of cytotoxic agents, MC diverges by embracing a continuous administration of chemotherapeutics at doses that reside significantly below the threshold of maximum tolerated dose (MTD). Notably absent are the pronounced interdose intervals, as this therapeutic approach seeks to maintain an unwavering presence of pharmacological agents within the patient's system.^2^, ^3^ Initially, the prevailing notion posited MC as primarily impeding angiogenesis. However, contemporary understanding underscores its multifaceted mechanisms, encompassing not only anti‐angiogenic effects but also anti‐proliferative attributes and immunomodulatory prowess.^3^, ^4^, ^5^, ^6^, ^7^ MC regimens were meticulously devised to optimize the antineoplastic potential of cytotoxic agents, thereby circumventing the recurrent challenges encountered with MTD chemotherapy.^8^ Notably, metronomic regimens exhibit a propensity for diminished toxicity, particularly with regards to grade 3 or 4 hematological, gastrointestinal, and infectious adversities. Such favorable toxicity profiles render MC a viable therapeutic option for patients who have undergone extensive prior treatments and now face the vexing conundrum of relapsed or refractory (R/R) diseases.^9^

The concept or philosophy of metronomics has been an integral part of pediatric oncology since its inception, with notable success achieved through the utilization of 6‐MP and methotrexate in the maintenance treatment of acute lymphoblastic leukemia. In the early 2000s, a substantial body of research focused on MC emerged, predominantly investigating its efficacy in the realm of brain tumors,^10^, ^11^, ^12^ alongside a handful of studies exploring its potential in refractory solid tumors.^13^, ^14^ In 2002, Sterba et al. pioneered the revelation of promising responses to metronomic temozolomide when combined with radiotherapy in pediatric patients with medulloblastoma. Subsequently, they embarked on a pilot study introducing a comprehensive four‐drug MC regimen for children confronting relapsed solid tumors across diverse histologies.^11^ In the second decade of the 2000s, a surge in research endeavors pertaining to MC for pediatric tumors has been documented. In parallel to findings from the preceding decade, these investigations encompass larger cohorts, thus consolidating the efficacy of metronomic therapeutic strategies. Presently, MC is being harnessed across a spectrum of pediatric malignancies, including leukemia, lymphoma, neuroblastoma, rhabdomyosarcoma, brain tumors, osteosarcoma, et al.

Neuroblastoma stands as the preeminent extracranial solid tumor afflicting the pediatric populace, emanating from cells within the sympathetic nervous system. Approximately half of afflicted patients manifest distant metastases at the time of initial diagnosis, with prevalent sites encompassing bone, bone marrow, and lymph nodes.^15^ Risk‐adapted treatment strategies are deployed accordingly upon the risk stratification of patients. Regrettably, the therapeutic efficacy in managing high‐risk patients and those grappling with R/R neuroblastoma remains suboptimal, underscored by a meager 5 year overall survival (OS) rate hovering around 20% for individuals navigating R/R patients.^16^ MC has been used in the treatment of neuroblastoma for over 20 years. In fact, the first MC in neuroblastoma article was published in 1999, although the term “metronomic chemotherapy” was not coined yet. Twenty children with R/R stage 4 neuroblastoma were treated with oral etoposide (50 mg/m^2^/d, in two or three divided doses) for 21 consecutive days followed by a 1 week rest.^17^ The disease control rate (complete response [CR]/partial response [PR]/SD) was 45% (9/20). Subsequent studies also suggested MC showed that this form of treatment appears to work in neuroblastoma. In this comprehensive review, we aim to provide readers with a thorough understanding of the application value and future directions of MC in neuroblastoma. Specifically, we have summarized the findings from clinical studies that have explored the use of MC in newly diagnosed and R/R neuroblastoma, thereby offering insights into its efficacy in these contexts. By examining the available evidence, we hope to shed light on the potential benefits of MC and identify areas for further investigation in the management of neuroblastoma.

## 2 DRUGS AND REGIMEN

MC is not defined by a single drug or cocktail but rather by the chronic administration of low doses of chemotherapy. It is important to note that no single regimen is likely to have universal efficacy, and the optimal combination of drugs for a specific tumor type remains unknown. In addition to chemotherapy, off‐patent nonchemotherapeutics such as vinblastine,^13^, ^18^ cyclophosphamide,^18^, ^19^, ^20^, ^21^ fenofibrate,^22^, ^23^ thalidomide,^23^, ^24^ celecoxib,^13^, ^18^, ^23^, ^25^ valproic acid,^21^, ^26^ and methotrexate^18^, ^21^, ^27^, ^28^ are used either in combination with or without chemotherapy in metronomic regimens. However, the most suitable combination regimen for neuroblastoma patients remains an unanswered question, as the MC regimens employed in studies vary. All of the MC components mentioned in this review are summarized in Table 1.

**TABLE 1:** Current used agents for neuroblastoma MC.

Drug	Common dose range	Common dose frequency
Cyclophosphamide	25–50 mg/m^2^ or 2.5 mg/kg	Days 1–21, followed by a 1‐week period of rest
Etoposide	25–50 mg/m^2^	Days 1–21, followed by a 1‐week period of rest
Celecoxib	100 mg, divided bid (<20 kg)	Daily
	200 mg, divided bid (20–50 kg)	
	400 mg, divided bid (>50 kg)	
Vinblastine	3 mg/m^2^	Weekly
Methotrexate	10–15 mg/m^2^	Twice a week for 3–4 weeks
Vincristine	1.5 mg/m^2^	Weekly for 4 weeks
Vinorelbine	25–60 mg/m^2^	Weekly for 3 weeks
Topotecan	1.4 or 0.22 mg/m^2^	Days 1–5 or daily
Thalidomide	3–24 mg/kg	Days 1–21 or daily
Fenofibrate	90–100 mg/m^2^	Daily
Valproic acid	20 mg/kg	Daily
Isotretinoin	100 mg/m^2^	Days 1–14, followed by a 2‐week period of rest
Temozolomide	30 mg/m^2^	Daily
Pazopanib	160 mg/m^2^	Daily since day 2
Zoledronic acid	2.4 mg/m^2^	Day 1
Cholecalciferol	300 kU/m^2^	Day 1
Vitamin D3	1500 U/m^2^	Days 1–78
Bevacizumab	10 mg/kg	Every 14 days
Nivolumab	3 mg/kg/dose	Days 1, 15

In the context of MC for neuroblastoma and other R/R solid tumors, oral cyclophosphamide and oral etoposide have emerged as the most frequently utilized drugs. These agents are often administered alone or in combination with nonchemotherapeutic agents like celecoxib and thalidomide. The four‐drug regimen consisting of oral cyclophosphamide, oral etoposide, celecoxib, and thalidomide has been extensively employed in various trials involving a heterogeneous mix of R/R solid tumors, including neuroblastoma. Such combination therapies represent a significant area of exploration in MC, aiming to enhance treatment outcomes in these challenging patient populations.^12^, ^24^ A recent study conducted by Sun investigated the benefits of MC in high‐risk neuroblastoma patients, specifically those who did not receive autologous stem cell transplantation (ASCT) or GD2 antibody therapy. The study utilized a combination of oral cyclophosphamide, vinorelbine, etoposide, topotecan, and celecoxib. The results showed promising outcomes, with a 3 year event‐free survival (EFS) rate of 42.5 ± 5.1% and an OS rate of 71.1 ± 4.7%. Importantly, the toxicities associated with MC were mild.^29^ These findings suggest that oral MC has the potential to improve survival outcomes in high‐risk neuroblastoma patients who achieve CR/very good partial response/PR without the need for ASCT or anti‐GD2 antibodies therapy. Furthermore, the study highlights the growing interest in exploring rational combinations of oral agents with immunotherapy approaches, which holds promise for further improving treatment outcomes in neuroblastoma.^30^, ^31^, ^32^

The MC regimen for neuroblastoma has shown variations across different studies, making it difficult to determine the most suitable combination for neuroblastoma patients. In the NB90 and NB97 trials, the MC regimen involved administration for only 5–8 days per month with a long drug‐free interval.^33^, ^34^ However, recent investigations have embraced a prolonged treatment duration, with MC drugs being administered for a minimum of 20 days per month.^29^ Intriguingly, a truncated drug‐free interval, coupled with the inclusion of vinorelbine, etoposide, and celecoxib in the MC regimen, has yielded the most favorable outcomes.

In the context of R/R neuroblastoma, the MC regimen exhibits even greater diversity. Both single‐agent and multiple drug combinations have been reported in the literature. Among the different drugs used in MC, cyclophosphamide stands out as the most commonly employed agent, as evidenced by existing reports. In addition to chemotherapeutic agents, non‐chemotherapeutic agents are often incorporated into MC regimens. Among these agents, celecoxib emerges as the most frequently used non‐chemotherapeutic agent in MC for R/R neuroblastoma. These findings highlight the wide range of options available when designing MC regimens for R/R neuroblastoma patients, with cyclophosphamide serving as a prominent choice for chemotherapeutic intervention and celecoxib acting as a frequently utilized non‐chemotherapeutic component.

As of now, the MC regimen for R/R neuroblastoma predominantly revolves around the utilization of cyclophosphamide, either in combination or in alternation with etoposide, serving as its steadfast foundation. *Vinca alkaloids* are often added to the regimen, while the use of methotrexate is less frequent. Notably, since 2020, there has been a growing trend of incorporating immunotherapy agents, such as nivolumab, and targeted therapy agents, such as pazopanib, into MC regimens for neuroblastoma. Despite these developments, determining the optimal MC regimen for R/R neuroblastoma remains a formidable task. However, the combination of cyclophosphamide, etoposide, vinca alkaloids, and celecoxib appears to be the current mainstream MC regimen. The exploration of incorporating novel targeted therapies or immunotherapies into the existing MC framework necessitates further investigation through future clinical trials.

## 3 THE ROLE OF MAINTENANCE THERAPY FOR NEWLY DIAGNOSED HIGH‐RISK NEUROBLASTOMA

The treatment approach of high‐risk neuroblastoma involves three main phases: induction, consolidation, and maintenance. In the induction phase, ongoing clinical trials are exploring the augmentation of conventional chemotherapy regimens through the inclusion of nonchemotherapeutic agents. These agents encompass targeted radiopharmaceuticals like 131I‐metaiodobenzylguanidine, ALK inhibitors, and anti‐GD2 monoclonal antibodies. The primary objective behind incorporating these agents into the chemotherapy backbone is to assess their potential in improving the CR rate and overall treatment outcomes.^35^ During the consolidation phase, the utilization of tandem ASCT has been proposed and has demonstrated a significantly higher 3 year EFS.^36^ This involves administering two consecutive ASCT procedures to further eradicate residual disease and enhance long‐term disease control. In the maintenance phase, immunotherapy with the combination of anti‐GD2 monoclonal antibodies and isotretinoin has emerged as the standard therapy.^37^ While MC has shown promise in certain neuroblastoma settings, its applicability within standard treatment protocols for newly diagnosed high‐risk neuroblastoma patients is currently limited.

MC has emerged as a potential approach for maintenance therapy in neuroblastoma, with cyclophosphamide being the most commonly used chemotherapeutic agent in this context. Previous studies have demonstrated that even tumors resistant to conventional chemotherapy may exhibit responses to metronomic cyclophosphamide regimens, likely due to both anti‐angiogenic and direct antitumor effects.^38^ In a randomized controlled trial conducted by Frank Berthold, the efficacy of oral cyclophosphamide as maintenance chemotherapy was compared to ASCT. The results showed that maintenance cyclophosphamide was inferior to ASCT, with 3 year EFS rates of 31% and 47%, respectively. However, there was no significant difference in 3 year OS rates, which were 53% in the maintenance cyclophosphamide arm and 62% in the ASCT arm.^33^ Another study by Thorsten Simon investigated the long‐term outcomes of high‐risk neuroblastoma patients after immunotherapy with antibody ch14.18 or oral MC. The results revealed that ch14.18 consolidation improved outcomes compared to no consolidation. However, no significant difference was found between MC and ch14.18‐treated patients.^34^ A recent retrospective study aimed to assess the effectiveness of 1 year MC in newly diagnosed stage 4 neuroblastoma patients. The findings revealed that MC continues to demonstrate more favorable outcomes when compared to the absence of maintenance therapy.^29^ As a result, MC can serve as a viable alternative when ASCT or immunotherapy is not feasible or limited by factors such as availability or cost. The favorable toxicity profile of MC further enhances its appeal as a pragmatic choice for maintenance therapy. However, additional trials or systematic reviews are required to further solidify its role in clinical practice.

It is worth noting that, as of now, there have been no clinical trials that have specifically examined the effectiveness of MC in combination with contemporary treatments. However, preclinical studies have explored the efficacy of combining MC with ALK inhibitors or mTOR inhibitor (Torin‐2).^39^, ^40^ These studies have demonstrated that the combination therapy yields stronger therapeutic effects. Nevertheless, additional research is required to determine the clinical applicability of these findings. MC may potentially serve as a foundation for novel small molecules or as a component of intensified treatment regimens in the future, further emphasizing its potential role in neuroblastoma management.

## 4 THE ROLE OF METRONOMIC CHEMOTHERAPY IN R/R NEUROBLASTOMA PATIENTS

While there have been notable advancements in the treatment of high‐risk neuroblastoma over the past few decades, it remains concerning that a significant proportion of children still experience relapse or fail to respond to initial therapy. The survival rates for those with R/R high‐risk neuroblastoma are dismal, with 4 year progression‐free survival and overall survival rates of only 6% and 20%, respectively.^16^ The median progression‐free survival in this population is a mere 6.4 months.^41^ Clearly, there exists a critical and urgent need for the development of more targeted therapeutic regimens that specifically address the unique characteristics of the tumor. Incorporating anti‐GD2 monoclonal antibodies along with the chemotherapeutic agents irinotecan and temozolomide has shown promise in achieving a more favorable response rate of 53%.^42^ Additionally, the use of difluoromethylornithine in high‐risk neuroblastoma patients has been approved, demonstrating improved EFS and OS rates compared to historical rate.^43^ Currently, there is an ongoing phase II study, ANBL1821, evaluating the combination of difluoromethylornithine with irinotecan, temozolomide, and dinutuximab in R/R neuroblastoma. However, it is important to note that there are no studies directly comparing the efficacy of MC with other treatment options for R/R neuroblastoma. Therefore, the evidence supporting the use of MC in this particular clinical scenario is limited, underscoring its restricted role in addressing the challenges associated with R/R neuroblastoma.

Table 2 provides a comprehensive summary of clinical trials investigating the use of MC in R/R neuroblastoma patients. It is worth noting that only three studies have specifically explored the potential benefits of MC in this context. In 1999, the introduction of oral etoposide in the treatment of R/R stage four neuroblastoma yielded promising results. Out of 20 patients, nine demonstrated a positive response, with one achieving CR, two showing PR, and six exhibiting SD.^17^ Another study investigated the efficacy of zoledronic acid (2–4 mg/m^2^, day 1) in combination with low‐dose cyclophosphamide (25 mg/m^2^/d, days 1–28) in 21 patients with R/R neuroblastoma. The results revealed that 1 patient achieved PR, 9 patients had SD, and 10 patients showed PD.^19^ Another study with 23 R/R neuroblastoma patients explored the use of a four‐drug combination MC regimen. This regimen consisted of oral cyclophosphamide (25 mg/m^2^/d, days 1–28), oral etoposide (25 mg/m^2^/d, days 1–21, administered every second month for four cycles only), vinblastine (3 mg/m^2^/d, days 1 and 15), and celecoxib (200 mg/m^2^ twice daily, days 1–28). The results demonstrated that four patients achieved CR, eight had PR, and nine exhibited SD. The survival outcomes of this group were compared to those of a matched cohort comprising 274 first‐relapsed neuroblastoma patients treated with intensive cyclic chemotherapy with curative intent. Interestingly, the 1 year secondary event‐free survival rates were 61.1% for the MC group and 35.4% in the control group, although this difference did not reach statistical significance (*p* = 0.204).^44^ MC is being employed as a cost‐effective and viable approach to rescue patients who have encountered relapse or are facing refractory neuroblastoma.

**TABLE 2:** Current MC clinical trials for relapsed/refractory neuroblastoma patients.

Study year	Disease	No of patients (NB/total)	Type of study	Metronomic chemotherapy regimen	Response	Refs
1999	Neuroblastoma	20/20	Phase 1/2	Etoposide (50 mg/m^2^, d1–21)	1 CR, 2 PR, 6 SD, 11 PD	17
2006	Recurrent/refractory solid tumors	3/33	Pilot	Celecoxib (250 mg/m^2^ divided bid)	No CR or PR	13
				Vinblastine (1 mg/m^2^ thrice a week) or cyclophosphamide (30 mg/m^2^)		
2011	Recurrent/refractory neuroblastoma	21/21	Phase 1	Cyclophosphamide (25 mg/m^2^, d1–28)	1 PR, 9 SD, 10 PD, 1 NA	19
				Zoledronic acid (2–4 mg/m^2^, d1)		
2011	Refractory tumors	1/12	Pilot	Cyclophosphamide (25 mg/m^2^, d1–21)	1 PD	27
				Methotrexate (15 mg/m^2^ twice a week, d21–42)		
2011	Refractory/relapsing tumors	1/16	Pilot	Celecoxib (<20 kg: 100 mg bid, 20–50 kg: 200 mg bid, >50 kg: 400 mg bid, d1–42)	1 PD	18
				Cyclophosphamide (30 mg/m^2^, d1–21)		
				Methotrexate (15 mg/m^2^, twice a week, d21–42)		
				Vinblastine (3 mg/m^2^, weeks 1–6)		
				Radiotherapy		
2012	Progressive/relapsed solid tumors	11/74	Retrospective	COMBAT I–III schedules combining variously: Temozolomide 30–60 mg/m^2^, d36–77	2 PR, 9 temporary responses or SD but soon PD	22
				Etoposide 25 mg/m^2^, d1–21 or d1–35		
				Celecoxib 200–400 mg/m^2^, d1–77 or 1–78		
				Cholecalciferol 300 kU/m^2^, d1		
				Vitamin D3 1500 U/m^2^, d1–78		
				Fenofibrate 100 mg/m^2^, d1–78		
				Isotretinoin 100 mg/m^2^, d1–14, 29–42, 57–70		
				Bevacizumab i.v. 10 mg/kg, every 14 days		
2012	Relapsed/refractory solid tumor	16/117	Phase II	Cyclophosphamide (25 mg/m^2^, d1–28)	1 PR, 2 SD, 13 PD	20
				Vinorelbine (25 mg/m^2^, d1, 8, and 15)		
2013	Refractory/advanced cancer	1/7	Pilot, prospective	Cyclophosphamide (25 mg/m^2^, d1–21)	1 PR	21
				Methotrexate (15 mg/m^2^ twice a week, d21–42)		
				Valporic acid (30 mg/kg)		
				Vincristine (1.5 mg/m^2^, d1, 8, 15, 22)		
2014	Recurrent/progressive cancer	3/101	phase II	Celecoxib (<20 kg: 100 mg bid, 20–50 kg: 200 mg bid, >50 kg: 400 mg bid)	2 SD, 1 PD	23
				Fenofibrate (90 mg/m^2^, max 200 mg/day)		
				Thalidomide (3–24 mg/kg, max 1000 mg/day)		
				Alternating cyclophosphamide/etoposide. (2.5 mg/kg, d1–21)/(50 mg/m^2^, d1–21)		
2016	Refractory/relapsed solid tumors	13/64	Phase II, prospective	Celecoxib (<20 kg: 100 mg bid, 20–50 kg: 200 mg bid, >50 kg: 400 mg bid, d1–42)	No specific value reported, 1‐year overall survival: 53.8%	25
				Cyclophosphamide (2.5 mg/kg, d1–21)		
				Methotrexate (15 mg/m^2^, twice a week, d21–42)		
				Vinblastine (3 mg/m^2^, weeks 1–6)		
				Radiotherapy		
2017	Recurrent high‐risk neuroblastoma	23/23	Phase 1/2	Celecoxib (200 mg/m^2^ bid, d1–28)	4 CR, 8 PR, 9 SD, 2 PD	44
				Cyclophosphamide (25 mg/m^2^ , d1–28)		
				Etoposide (25 mg/m^2^, d1–21, every second month)		
2017	Primary extracranial, nonhematopoietic solid malignant tumors that progress after at least two lines of chemotherapy	MC: 5/56 Control: 5/52	Phase III	Celecoxib (<20 kg: 100 mg bid, 20–50 kg: 200 mg bid, >50 kg: 400 mg bid)	No specific value	24
				Thalidomide (3 mg/kg, days 1–21)		
				Alternating cyclophosphamide/etoposide (2.5 mg/kg, d1–21)/(50 mg/m^2^, d1–21)		
2019	Relapsed/progressing extracranial solid tumors	18/44	Phase II	Celecoxib (<20 kg: 100 mg bid, 20–50 kg: 200 mg bid, >50 kg: 400 mg bid, d1–56)	4 SD, 14 PD	28
				Cyclophosphamide (30 mg/m^2^, d1–21)		
				Methotrexate (10 mg/m^2^ twice a week, d22–43)		
				Vinblastine (3 mg/m^2^, weeks 1–7)		
2020	Refractory/relapsing malignancies	24/98	Phase II, prospective	Cyclophosphamide (30 mg/m^2^, d1–21)	1 PR, 4 SD, 19 PD	26
				Etoposide (25 mg/m^2^, d1–21)		
				Valproic acid (20 mg/kg, d1–28)		
2021	Relapsed/refractory solid tumors	3/13	Phase II	Cyclophosphamide (25 mg/m^2^ bid, interval of 1 week)	3 PD	45
				Nivolumab (3 mg/kg/dose, d1, 15)		
				Radiotherapy		
2022	Relapsed/refractory solid tumors	7/30	Phase I	Topotecan (0.22 mg/m^2^, d1–28)	4 SD, 3 PD	32
				Pazopanib. (160 mg/m^2^, d2–28)		
2022	Tumors relapsing to conventional dose chemotherapy	1/6	Retrospective case series	Alternating cyclophosphamide/etoposide (75 mg/m^2^, d22–41)/(50 mg/m^2^, d1–21)	1 PR	46
				Sulindac/celecoxib (4 mg/kg)/(500 mg/m^2^)		

Abbreviations: CR, complete response; PR, partial response.

While MC shows great potential in addressing the challenges of R/R neuroblastoma, its efficacy remains relatively understudied due to the overshadowing presence of concurrent clinical trials, which complicate patient recruitment. The majority of clinical trials encompass a broader scope, enrolling pediatric patients with diverse R/R cancer types, including neuroblastoma among others. Consequently, the number of neuroblastoma cases included in these trials has been limited, with a mere 112 patients with R/R neuroblastoma being reviewed across the 14 trials.^13^, ^17^, ^18^, ^19^, ^20^, ^21^, ^22^, ^23^, ^24^, ^25^, ^26^, ^27^, ^28^, ^32^, ^44^, ^45^, ^46^

Excluding 23 patients from two studies where treatment responses were not specifically reported, a total of 89 patients were available for statistical analysis across the 14 trials. When combined with the three specific studies investigating the role of MC in R/R neuroblastoma, a cohort of 153 patients was eligible for prognostic evaluation. PD emerged as the predominant overall response observed among neuroblastoma patients undergoing MC, with 91 out of 153 patients experiencing PD. The incidences of PR or SD under MC remained infrequent, while CR was relatively rare, with only five patients achieving this outcome. Furthermore, the variation in drug combinations, schedules, and dosages across different studies adds significant complexity to the analysis. This variability hampers direct comparisons and comprehensive assessments of the overall effectiveness and outcomes associated with MC treatment in R/R neuroblastoma. To address this challenge, a harmonized MC protocol is therefore warranted to further investigate its role in treating R/R neuroblastoma. Additionally, in certain literature, detailed outcomes specific to neuroblastoma patients were not consistently reported. This inconsistency further contributes to the challenges in comprehensively understanding the impact and efficacy of MC within the context of neuroblastoma treatment. Standardized reporting guidelines for neuroblastoma clinical trials would enhance data transparency and facilitate more accurate comparisons and interpretations of treatment outcomes across studies.

The treatment of R/R neuroblastoma presents significant challenges in low‐ and middle‐income countries due to disparities in healthcare access. Many advanced treatment options, such as meta‐iodobenzylguanidine therapy, anti‐GD2 monoclonal antibodies, and ASCT, are difficult to access in these regions. Consequently, managing R/R neuroblastoma in these settings poses even greater difficulties. MC may play a crucial role in addressing these challenges, given its wide availability, relatively low costs, and minimal infrastructure requirements. The International Society of Pediatric Oncology (SIOP) has recognized MC as a potential palliative treatment for R/R neuroblastoma in low‐ and middle‐income countries.^47^ While MC offers advantages such as outpatient use and low toxicity, there are several drawbacks to consider. These include the lack of scientific evidence or treatment guidelines specific to MC, issues related to drug availability and affordability, and concerns regarding patient acceptance and compliance.^48^ The current literature highlights the limited accessibility of ASCT, anti‐GD2 immunotherapy, and other targeted therapies as the main driving force behind the utilization of MC in the treatment of both newly diagnosed and R/R neuroblastoma. In resource‐limited regions, MC often serves as a common and vital alternative to ASCT or immunotherapy. However, to address this unmet need, it is crucial to establish standardized MC protocols and conduct clinical trials that can generate high‐level evidence. These essential steps will help bridge the gap and provide effective treatment options for neuroblastoma patients in resource‐limited settings.

## 5 THE ROLE OF METRONOMIC CHEMOTHERAPY IN PALLIATIVE NEUROBLASTOMA PATIENT

Although limited, MC has been employed in pediatric cancer patients as a means of palliative care, primarily due to its affordability and minimal toxicities.^49^ However, no published studies have discussed MC as a palliative treatment in detail. While a previous randomized trial did not demonstrate a significant extension of progression‐free survival in progressive pediatric solid malignant tumors, it did reveal that MC was associated with an improved quality of life during palliative cancer care for children.^50^ Given that PD is the predominant overall response observed in MC therapy for R/R neuroblastoma, the primary focus lies in determining whether MC is associated with an enhanced quality of life. Currently, the available evidence is limited, with only one MC trial, Metro‐SMHOP 01, including neuroblastoma patients and assessing quality of life as an endpoint. Within this cohort of 98 patients, 24 were diagnosed with neuroblastoma. The median initial Karnofsky/Lansky evaluation percentage was 50%, which increased to 70% in the final assessment. Notably, 15 out of the 98 patients exhibited improved Karnofsky/Lansky scores.^26^ While the evidence supporting MC as a palliative treatment for neuroblastoma patients remains limited, a survey investigating physicians' perspectives on palliative care for neuroblastoma revealed that 52% considered MC a viable treatment option.^51^ Further studies that prioritize quality of life assessment as the primary endpoint are necessary to ascertain the role of MC in the palliative setting for neuroblastoma patients.

## 6 TOXICITY OF METRONOMIC CHEMOTHERAPY

Despite variations in reported efficacy across different study cohorts, drug regimens, and tumor types, collective clinical trial data consistently underscore the favorable tolerability profile of MC, either as a standalone modality or in combination therapies. Predominantly, myelosuppression emerges as the main treatment‐related adverse event, although most hematologic toxicities are mild, grades 3/4 toxicities are not uncommon, and grade 3/4 non‐hematologic toxicities remain infrequent.^17^, ^25^, ^29^, ^33^, ^44^ Furthermore, certain toxicities such as elevations in aminotransferases, lipase, hypocalcemia and hypophosphatemia have been noted when MC is co‐administered with other agents like pazopanib,^32^ thalidomide,^23^ zoledronic acid^19^ and bevacizumab.^22^ The combination of MC with bevacizumab^22^ or nivolumab^45^ appears to exert no discernible amplification of individual treatment toxicities. Nevertheless, the available data remain circumscribed, precluding definitive assertions regarding the tolerability of these combinatorial approaches. Overall, MC manifests as a therapeutic paradigm characterized by minimal toxicity burdens, thereby conferring substantive clinical benefits and ameliorating the quality of life for patients with advanced and/or relapsed malignancies.

Indeed, while MC holds promising therapeutic potential for pediatric populations, it is essential to approach its use with caution and careful consideration of potential risks and concerns. Absolutely, the intricate balance of angiogenesis plays a pivotal role in both normal physiological growth and the pathogenesis of tumors, including neuroblastoma. As MC aims to disrupt tumor‐associated neo‐angiogenesis, it's crucial to consider its potential impact on physiological angiogenic processes in developing children. Additionally, concerns arise regarding the potential accumulation of high cumulative doses of anticancer agents with protracted exposure to MC. This accumulation raises apprehensions about the potential for increased toxicity and predisposition to secondary pathologies in affected individuals. For instance, the administration of elevated cumulative doses of etoposide^52^ or temozolomide^53^ has been linked to the onset of secondary leukemias. In Robison's study, an ependymoma patient who underwent a 21 month treatment with a 5‐drug regimen (celecoxib/fenofibrate/thalidomide/cyclophosphamide/etoposide) and subsequently developed t (8; 21) acute myelogenous leukemia, 26 months after initiating the study.^23^ Although such secondary malignancies have not been reported in neuroblastoma patients undergoing MC, meticulous surveillance of pediatric recipients is imperative in forthcoming investigations.

## 7 POTENTIAL BIOMARKERS FOR METRONOMIC CHEMOTHERAPY IN NEUROBLASTOMA PATIENTS

While the collective efficacy of MC in managing R/R neuroblastoma appears somewhat constrained, select literature suggests a spectrum of responses, ranging from CR in certain cases^17^, ^44^ to the maintenance of PR or SD in others under MC.^19^, ^20^, ^21^, ^22^, ^23^, ^26^, ^28^, ^32^, ^46^ Consequently, the identification of prognostic factors or biomarkers within the cohort of neuroblastoma patients undergoing MC assumes paramount importance. The quest for robust and insightful biomarkers—embracing diagnostic, predictive, and prognostic realms—becomes imperative. Such biomarkers hold the promise of discerning which patients are poised to derive optimal benefits from MC and facilitating the ongoing monitoring of treatment activity. This necessity is accentuated by the gradual and nuanced action of these therapies, which may not promptly elicit tumor regression.

The COMBAT study posited that neuroblastoma exhibiting a slower growth trajectory may exhibit greater susceptibility to MC compared to their rapidly proliferating counterparts, particularly those infiltrating the bone marrow.^36^ This assertion underscores the potential import of tumor attributes, growth kinetics, and specific disease nuances as determinants of MC treatment responsiveness. The quest for additional surrogate markers becomes imperative to steer patient selection and prognosticate responses among neuroblastoma patients undergoing MC. Noteworthy investigations have spotlighted the role of HIF‐1α expression^54^ and factors implicated in MC resistance (e.g., cathepsin B, ANXA3, and TXNDC5)^4^, ^55^ as potential biomarkers. Pramanik et al. conducted a seminal inquiry probing vascular endothelial growth factor (VEGF) and thrombospondin‐1 (THBS1) as candidate biomarkers for MC in progressive pediatric solid malignancies.^49^ Within this study cohort, six neuroblastoma patients received MC while an equivalent number were administered a placebo. Additionally, cytokines such as basic fibroblast growth factor (bFGF), endostatin, and the immune microenvironment have been scrutinized for MC efficacy assessment in diverse pediatric cancer contexts.^12^, ^23^, ^45^, ^56^, ^57^ However, some of these investigations omitted neuroblastoma patients, yielding contradictory findings with certain studies advocating for their predictive role while others refute it. Furthermore, disparate drug combinations, diverse underlying pathologies, and inconsistent study outcomes compound the challenge of arriving at definitive conclusions. Hence, there is a compelling impetus to integrate novel biomarkers such as cell‐free DNA (cfDNA), circulating tumor cells, and next‐generation sequencing information into forthcoming investigations.

## 8 CONCLUSION AND FUTURE DEVELOPMENTS

Given its cost‐effectiveness, accessibility, and favorable toxicity profile, MC holds substantial importance in the management of neuroblastoma. For newly diagnosed high‐risk neuroblastoma patients, MC remains a critical consideration, particularly for individuals who lack access to or cannot afford standard ASCT or immunotherapy in resource‐constrained regions. In the context of R/R neuroblastoma, MC represents a viable option for patients who may not tolerate intensified treatments due to extensive prior therapies, lack access to immunotherapy, or simply require palliative care. Nevertheless, the optimal MC regimen has yet to be established. Future preclinical and clinical studies should elucidate the most efficacious agents contingent upon tumor histology, biomarkers and prognostic determinants to ascertain the ideal amalgamation of therapeutic compounds, delineate precise dosages for singular or synergistic administration, and refine the temporal aspects of drug delivery. Additionally, optimization of treatment duration and cessation protocols remains paramount for therapeutic advancement.

It remains essential to undertake exploratory endeavors aimed at identifying potential biomarkers or prognostic determinants to steer MC treatment strategies and to explore the feasibility of integrating innovative molecular modalities into the current MC framework, thereby enhancing its therapeutic efficacy.

## AUTHOR CONTRIBUTIONS

**Yi Jiang:** Data collecting; literature search; writing‐original draft; writing‐reviewing and editing. **Chengjun Xi:** Data collecting; literature search; writing‐original draft; writing‐reviewing and editing. **Chao Yang:** conceptualization; supervision; writing‐reviewing and editing.

## CONFLICT OF INTEREST STATEMENT

The authors have no relevant financial or non‐financial interests to disclose.

## ETHICS STATEMENT

Due to the nature of this review, the ethics review was waived by the institution review board.

## Data Availability

Data sharing not applicable to this article as no datasets were generated or analyzed during the current study.
